# CRL3 E3 ligase regulates glutamine and cystine metabolisms

**DOI:** 10.1093/procel/pwae051

**Published:** 2024-09-13

**Authors:** Qiyin Zhou, Zhijian Li, Yi Sun

**Affiliations:** Cancer Institute (Key Laboratory of Cancer Prevention and Intervention, China National Ministry of Education), The Second Affiliated Hospital, Zhejiang University School of Medicine, Hangzhou 310009, China; Institute of Translational Medicine, Zhejiang University School of Medicine, Hangzhou 310029, China; Cancer Center, Zhejiang University, Hangzhou 310058, China; Zhejiang Provincial Clinical Research Center for Cancer, Hangzhou 310009, China; Institute of Translational Medicine, Zhejiang University School of Medicine, Hangzhou 310029, China; Cancer Center, Zhejiang University, Hangzhou 310058, China; Cancer Institute (Key Laboratory of Cancer Prevention and Intervention, China National Ministry of Education), The Second Affiliated Hospital, Zhejiang University School of Medicine, Hangzhou 310009, China; Institute of Translational Medicine, Zhejiang University School of Medicine, Hangzhou 310029, China; Cancer Center, Zhejiang University, Hangzhou 310058, China; Zhejiang Provincial Clinical Research Center for Cancer, Hangzhou 310009, China; Research Center for Life Science and Human Health, Binjiang Institute of Zhejiang University, Hangzhou 310053, China

Tumorigenesis hinges on the reprogramming of cellular metabolisms to meet the requirements of intracellular biomass, characterized by the acquisition of nutrients from the environment as a common feature (Pavlova and Thompson, 2016). Moreover, to fine-tune and support the cellular activities essential for growth and survival, tumor cells integrate multiple metabolic pathways to regulate nutrient availability upon metabolic reprogramming in tumor microenvironment (Lobel et al., 2023).

Glutamine is one of the principal nutrients and the most abundant plasma amino acids, and cancer cells are highly dependent on it for proliferation (Yang et al., 2017). Cancer cells uptake glutamine through its transporters and use it as both nitrogen and carbon sources for biosynthetic processes and anabolic metabolites. During the glutaminolysis, glutamine intermediate glutamate can be metabolically converted into α-ketoglutarate (α-KG) and then it enters into the tricarboxylic acid (TCA) cycle, or be utilized to produce glutathione (Lobel et al., 2023). Notably, to ensure the increased glutaminolysis, cancer cells secrete partial glutamate, in exchange of extracellular cystine, through its transporters, X_c_^−^ system, consisting of SLC7A11 and SLC3A2 (Muir et al., 2017; Timmerman et al., 2013). Imported cystine is converted to its reduced form cysteine, a proteinogenic amino acid that participates in protein synthesis, post-translational modification, redox maintenance, and ferroptosis (Koppula et al., 2021). Thus, cancer cells sustain high levels of glutamine metabolism by increasing glutamine uptake and concurrently orchestrating the secretion of its intermediate glutamate for cystine importation. Elucidation of the molecular mechanisms into how the glutamine–cystine uptakes are coordinately regulated would deepen our understanding of glutamine and cystine metabolisms in cancer cells, and provide a strategic rationale to target these metabolisms for effective cancer therapy.

Neddylation is one type of post-translational modifications that attaches NEDD8, a small ubiquitin-like protein, onto a lysine residue of the substrate protein to modulate its activity, subcellular localization, or function (Vogl et al., 2020). Neddylation is catalyzed by an E1 NEDD8-activity enzyme (NAE), an E2 NEDD8-conjugating enzyme, and an E3 neddylatin ligase (Zhang et al., 2024). Cullin family members, the scaffold component of cullin-RING ligase (CRL), are well-known physiological substrates of neddylation. A typical CRL E3 consists of a scaffold cullin with eight family members (cullins 1, 2, 3, 4A, 4B, 5, 7, and 9), an adaptor protein, a substrate-recognizing receptor, and a RING protein (RBX1 and RBX2/SAG) (Zhao and Sun, 2013). Cullin neddylation activates CRL, the largest family of ubiquitin E3 ligases, thereby regulating many important physiological and pathological processes by targeting cellular proteins for proteasome degradation (Zhou et al., 2018).

It has been shown that NEDD8 and neddylation enzymes are overexpressed in a variety of human cancers, which is associated with poor survival of cancer patients. A variety of preclinical studies have validated that neddylation is an attractive anticancer target (Zhou et al., 2018). In fact, MLN4924, also known as pevonedistat, the first-in-class inhibitor of NAE, is currently advanced into a number of phases I–III clinical trials as a single agent or in combination with chemo drugs (Zhang et al., 2024). By blocking neddylation to inactivate CRLs, resulting in accumulation of many tumor suppressor substrates, MLN4924 has been shown to induce cell cycle arrest, apoptosis, autophagy, and senescence in a variety of cancer cells (Zhao and Sun, 2013). Our earlier studies showed that MLN4924 markedly alters global metabolic profiling, including inhibition of mitochondrial function and promotion of glycolysis (Zhou et al., 2019). In the past two years, we determined whether and how neddylation regulates other metabolic pathways in addition to the energy metabolism.

We treated breast cancer cells with MLN4924 and identified the differentially expressed metabolites through mass-spectrometry analysis in cell extracts and culture supernatants, followed by GO analysis to define the altered metabolic pathways (Zhou et al., 2022). In both cell extracts and culture supernatants, the glutamine metabolism was markedly changed. Specifically, MLN4924 treatment reduced glutamine levels in culture media, indicating an increased glutamine uptake. We also found an elevated glutamate levels in culture media, which are typical features of increased glutamine metabolism (Muir et al., 2017), as a result of possible activation of both transporters for glutamine import and glutamate export. Thus, neddylation inactivation promotes glutamine metabolism and possibly affects X_c_^−^ system, which is an antiporter that exports intracellular glutamate coupled with importing extracellular cystine.

We first focused on the underlying mechanism by which MLN4924 regulates glutamine metabolism (Zhou et al., 2022). Screening for possible accumulation of several primary transporters and enzymes involved in glutamine metabolism, we found that glutamine transporter ASCT2 was accumulated at the highest level upon MLN4924 treatment. It is well-established that neddylation inhibition by MLN4924 inactivates CRL activity and causes the accumulation of CRL substrates (Zhao and Sun, 2013), we hypothesized that ASCT2 could be a substrate of CRL. To this end, we used siRNA-based knockdown approach targeting five cullins (cullins 1–5), and found that knockdown of cullin-3, but not of other cullins, caused ASCT2 accumulation. Follow-up study revealed that MLN4924 inactivates CRL3^SPOP^ E3 ligase, leading to accumulation of ASCT2. We next defined how CRL3^SPOP^ promotes ASCT2 ubiquitylation, and found that casein kinase 1δ (CK1δ) phosphorylates serine residues within the SPOP binding consensus motif of ASCT2, a required process for substrate binding to SPOP (An et al., 2015), to facilitate the SPOP-ASCT2 binding and subsequent K48-linked ASCT2 polyubiquitylation. Interestingly, the SPOP-ASCT2 axis is subject to glutamine regulation. It is known that SPOP has to form a dimer to bind to its substrate for targeted ubiquitylation and degradation (Zhuang et al., 2009). Under glutamine deprivation condition. G protein-coupled receptor kinase 2 (GRK2) is induced to phosphorylate SPOP on the Ser^222^ residue, which disrupts its dimerization, and triggers its self-ubiquitylation and degradation, leading to inactivation of CRL3^SPOP^ E3 ligase, and subsequent accumulation of its substrate, ASCT2, leading to enhanced glutamine uptake (Zhou et al., 2022).

Biologically, both *in vitro* and *in vivo* experiments showed that ASCT2 has growth-promoting activity, whereas SPOP has anti-proliferation activity. Clinically, tumor tissues have higher levels of ASCT2, but lower levels of SPOP in breast cancer tissues, as compared to adjacent normal tissues. More importantly, high SPOP/low ASCT2 predicts a better, whereas low SPOP/high ASCT2 predicts a worse survival of breast cancer patients. Furthermore, in paired breast normal and tumor tissues from the same patients, tumor tissues have lower glutamine content than that of the normal tissue, suggesting enhanced glutamine metabolism in tumors. Thus, high levels of ASCT2 coupled with low levels of SPOP would increase glutamine transport and subsequent metabolism (Zhou et al., 2022).

Therapeutically, it is logical to combine MLN4924 with ASCT2 inhibitor for enhanced anti-cancer efficacy, given that neddylation inactivation increases glutamine uptake and metabolism through ASCT2 to sustain the growth of breast cancer cells. Indeed, MLN4924 coupled with V-9302, an ASCT2 inhibitor, significantly suppresses growth of breast cancer cells more effectively than either compound used alone, both *in vitro* and *in vivo*. Therefore, this study provides a rationale for a drug combination approach in cancer therapy (Zhou et al., 2022).

Given that environmental cystine and X_c_^−^ system actively orchestrate glutaminolysis (Muir et al., 2017; Timmerman et al., 2013), we hypothesized that X_c_^−^ system may coordinate neddylation-regulated glutamine metabolism. To this end, we found that in the culture supernatant cystine levels decreased upon MLN4924 treatment, along with an increased level of glutamate, as shown in our previous study (Zhou et al., 2022), indicating the enhanced cystine uptake through activated X_c_^−^ system. We subsequently found that between SLC7A11 and SLC3A2, two components of X_c_^−^ system, SLC7A11 was the one which accumulated substantially upon MLN4924 treatment, which is likely responsible for enhanced cystine uptake (Zhou et al., 2024).

Among cullins 1–5, knockdown of only cullin-3 caused accumulation of SLC7A11, accompanied by increased cystine uptake, consistent with our earlier observation that cullin-3 knockdown increased glutamate levels in the culture media (Zhou et al., 2022). We next screened 13 potential receptor proteins of CRL3 and identified KCTD10, whose knockdown increased SLC7A11 levels in multiple cell lines. KCTD10 indeed binds to the cytoplasmic N-terminal domain of SLC7A11 with its C-terminal domain and promotes SLC7A11 polyubiquitylation via the K48 linkage for proteasome degradation. Thus, CRL3^KCTD10^ is a *bona fide* E3 ligase for SLC7A11 (Zhou et al., 2024).

As ubiquitylation is a reversible process dynamically regulated by both E3 ligase and deubiquitylase (DUB), we went on to screen a battery of DUBs, and identified USP18 as a SLC7A11 DUB, responsible for SLC7A11 deubiquitylation and stabilization. Specifically, USP18 binds to again the cytoplasmic N-terminal domain of SLC7A11 via its internal domain (AA 51–150) and stabilizes SLC7A11 by extending its protein half-life (Zhou et al., 2024).

We next defined the physiological conditions that coordinately regulated the levels of SLC7A11, KCTD10, and USP18, and found that deprivation of cystine, not glutamine nor glucose was the only one doing so. Specifically, cystine deprivation increases the levels of both SLC7A11 and USP18 but decreases KCTD10 levels, which is reversible by cystine addition. Likewise, cystine deprivation increases the SLC7A11 and USP18 interaction and enhances SLC7A11 deubiquitylation by USP18, whereas decreases the SLC7A11 and KCTD10 interaction and reduces SLC7A11 polyubiquitylation by KCTD10. Finally, USP18 knockdown or KCTD10 overexpression decreases SLC7A11 accumulation induced by cystine deprivation. Together, it appears that environmental cystine levels regulate the interaction among SLC7A11, KCTD10, and USP18, and cystine starvation ensures high levels of SLC7A11 for cystine uptake by downregulating KCTD10 and upregulating USP18. Biologically, USP18 knockdown suppresses, whereas KCTD10 knockdown promotes growth of breast cancer cells via reducing or increasing SLC7A11, respectively. Thus, SLC7A11 is the hub for KCTD10 and USP18 to regulate cell growth (Zhou et al., 2024).

SLC7A11 has been well established as a key inhibitor of ferroptosis (Stockwell, 2022; Zhang et al., 2022). We then investigated whether, via modulating SLC7A11 levels, KCTD10 or USP18 would regulate ferroptosis. Using various canonical approaches, including cystine deprivation and ferroptosis inducers or inhibitors, we confirmed that KCTD10 knockdown inhibits ferroptosis, which is mitigated by SLC7A11 knockdown. Likewise, USP18 knockdown increases ferroptosis, which is rescued by ferroptosis inhibitors or ectopic SLC7A11 expression. Collectively, these results demonstrate that KCTD10 and USP18 as novel upstream regulators of SLC7A11, regulate ferroptosis by targeting SLC7A11 (Zhou et al., 2024).

In clinical breast cancer samples, we found elevated levels of SLC7A11 and USP18, and reduced levels of KCTD10. Moreover, the levels of SLC7A11 were positively correlated with USP18 but negatively correlated with KCTD10. Thus, low levels of KCTD10 coupled with high levels of USP18 would maintain elevated levels of SLC7A11 to enhance cystine uptake and subsequent metabolism, which could contribute to cystine addiction as often seen in cancer cells, which facilitates the spatial heterogeneity in cystine metabolism (Bonifacio et al., 2021). Therapeutically, inhibition of tumor growth both *in vitro* or *in vivo* was maximally observed when MLN4924 was combined with SLC7A11 inhibitor Erastin (for *in vitro*) or IKE (for *in vivo*) assay. Thus, simultaneously targeting neddylation and cystine transporter appears to be a viable strategy for anti-cancer therapy (Zhou et al., 2024).

In summary, our studies showed that the same CRL3 E3 ligase, via two different substrate-recognizing subunits, controls the transports of glutamine (via SPOP for targeting ASCT2) and cystine (via KCTD10 for targeting SLC7A11). By inactivating CRL3, MLN4924 would activate both transporters to enhance intracellular levels of both glutamine and cystine, thus orchestrating glutaminolysis (Fig. 1). On one hand, activated ASCT2 enhances glutamine uptake and metabolism and confers a survival advantage for cancer cells. Combining of MLN4924 with ASCT2 inhibitor could effectively block the enhanced glutamine metabolism and inhibit cancer cell growth. On the other hand, activated SLC7A11 enhances cystine uptake to facilitate cancer cell growth. Moreover, USP18 is a DUB that is induced upon cystine deprivation to stabilize SLC7A11 for enhanced cystine uptake. Co-targeting neddylation and cystine transport with MLN4924 and IKE remarkably suppress cancer cell growth. Together, our study demonstrates mechanistically how neddylation-CRL3 regulates the uptake of glutamine and cystine, from the tumor environment to fine-tune cancer cell growth. Combinational targeting neddylation and glutamine-cystine transporters would significantly enhance the efficacy of anti-cancer therapies. It is also conceivable that these two regulatory mechanisms could potentially affect each other. For example, targeted inhibition of ASCT2 via V9302 would reduce glutamine uptake, and intracellular glutamate levels, thereby decreasing cystine uptake, whereas targeted inhibition of SLC7A11 via erastin or IKE would increase cellular level of glutamate, potentially reducing glutamine uptake through ASCT2 via a feedback mechanism. Future studies should be directed to investigate the cross-talk between these two transporters under physiological and pathological stressed conditions.

**Figure 1.**
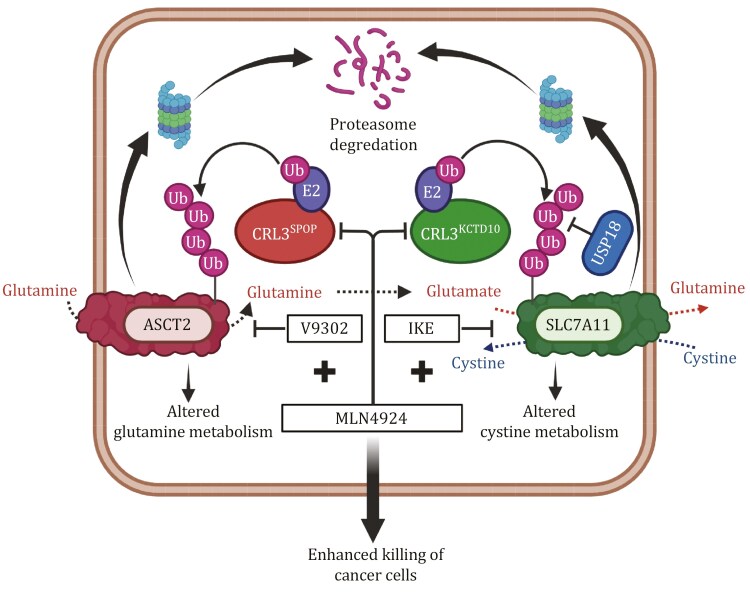
Neddylation-CRL3 E3 ligase regulates the uptake of glutamine and cystine. MLN4924 inactivates CRL3^SPOP^ ligase and causes ASCT2 accumulation, leading to enhanced glutamine uptake and metabolism. Simultaneously, it also inactivates CRL3^KCTD10^ E3 ligase, which coordinates with USP18 to stabilize SLC7A11, leading to enhanced cystine uptake and ferroptosis inhibition. The combination of MLN4924 and ASCT2 inhibitor V-9303 or SLC7A11 inhibitor IKE effectively suppresses tumor growth with enhanced cancer cell killing. The figure is created at Biorender.com.

## Data Availability

Not applicable.
